# When to change needles during neuromodulator injections—An electron‐microscopy investigation into needle tip deformation

**DOI:** 10.1111/jocd.16506

**Published:** 2024-11-05

**Authors:** Lisa Akintilo, Jeremy B. Green, Joely Kaufman, Bahar Ghane‐Motlagh, David L. Freytag, Konstantin Frank, Michael Alfertshofer, Sebastian Cotofana

**Affiliations:** ^1^ Skin Associates of South Florida and Skin Research Institute Coral Gables Florida USA; ^2^ BioNIUM Nanofabrication Facility University of Miami Miami Florida USA; ^3^ Department for Hand, Plastic and Aesthetic Surgery Ludwig – Maximilian University Munich Munich Germany; ^4^ Department of Plastic, Hand and Reconstructive Surgery University Hospital Regensburg Regensburg Germany; ^5^ Department of Dermatology Erasmus University Medical Center Rotterdam The Netherlands; ^6^ Centre for Cutaneous Research Blizard Institute, Queen Mary University of London London UK

**Keywords:** botulinum toxin, electron microscopy, needle injections, needle tip deformation, neuromodulator

## Abstract

**Background:**

Neuromodulator injections are minimally invasive procedures performed across the globe. Despite their ubiquity, there is a dearth of information on whether and how needle tips used for neuromodulator procedures are deformed after repeated injections.

**Objective:**

We investigated the magnitude of needle tip deformation following sequential injection passes (3×, 5×, and 10×) during facial neuromodulator injections with three commonly used needle sizes (30G, 31G, and 32G).

**Methods:**

Neuromodulator was administered for four different aesthetic indications. Each collected needle was mounted and imaged in a Philips XL‐30 Scanning electron microscope. Images were processed using ImageJ photo analysis software.

**Results:**

Forty‐five needle tips were investigated. When comparing the facial regions of interest, a statistically significant difference in deformation percentage was found when injecting 10× (*p* = 0.044) with greatest damage after injecting the glabella (38.4%), followed by lateral canthus (27.9%), forehead (27.5%), and midface (23.1%). Independent of facial region targeted, the mean percentage of needle deformation at 3× was 14.8%, at 5× 19.6%, and at 10× 29.2% with *p* < 0.001. Smaller needle size corresponded to smaller percentage of damage.

**Conclusion:**

Exchanging needles after more than five injection passes will minimize needle deformation and likely increase injection precision.

## 1 INTRODUCTION

Neuromodulator injections are minimally invasive procedures frequently performed by healthcare providers for various aesthetic indications, including facial wrinkles, facial contouring, skin quality improvement, and excessive sweating among others. In fact, injection of botulinum toxin type A for aesthetic indications is the number one minimally invasive cosmetic procedure in the United States, with an estimated 8 736 591 million treatments performed in 2022 according to the American Society of Plastic Surgeons.^1^

Anecdotally, after repeated injections with the same needle, more force is required to achieve cutaneous penetration. Typically, this is attributed to the needle becoming “dull” or less sharp. That increased force required to penetrate and pass through the dermis can potentially lead to decreased precision of injection when a specific tissue plane is targeted for placement.

It has previously been shown via scanning electron microscopy (EM) analysis^2^ that needle tips are deformed significantly after repeated soft tissue filler injections. To date, there is no published literature analyzing the effects of tissue introduction and re‐introduction on needles used for neuromodulator injections.

Determining whether or not repeated neuromodulator needles are damaged with prolonged use could change clinical practice by informing practitioners to change their needles more frequently. Neuromodulator manufacturers recommend use of a 30G–33G needle when administering treatment for aesthetic purposes^3^; no single specific gauge is recommended.

Perhaps by changing needles more frequently, less damage could occur to each needle tip and, as a corollary, less force would be required to allow the needle to pass into the skin, resulting in increased precision of neuromodulator treatments. This could potentially enhance patient comfort as well. For these reasons, we sought to provide insight into the often‐asked clinical question of when to change needles during neuromodulator injections.

## 2 MATERIALS AND METHODS

### 2.1 Study sample

Injection procedures were conducted in consecutive patients treated at Skin Associates of South Florida, Coral Gables, FL, USA by experienced injectors. After topical anesthesia was applied for approximately 20–30 min, patients were treated for facial rhytids and skin quality following standard operating procedures. The needles utilized in those injection procedures were collected and further investigated using EM imaging.

### 2.2 Needles investigated

A total of three differently sized needles were used and further investigated with EM imaging: 30G × 1/2 “(0.3 × 12 mm) (K‐Pack II® Terumo Europe, Leuven, Belgium), 31G × 5/16” (8 mm) (BD Ultra‐Fine™, Franklin Lakes, New Jersey, USA), 32G × 1/2 (0.26 × 13 mm) (TSK Laboratory, Tochigi, Japan). All needles were carefully collected after the injection procedures to ensure the tips only contacted patients during the neuromodulator procedure, with the exception of the two needles collected following typical neuromodulator reconstitution procedures. For each needle type, a new not previously utilized needle was additionally sent for EM imaging; this needle served as a reference.

### 2.3 Injection procedures

Informed consent for the aesthetic treatment was obtained by each patient as per standard practice. Neuromodulator was administered in four different aesthetic locations: forehead, glabella, lateral canthus, and midface/cheek. Treatment areas were prepped with alcohol, and patients were treated according to the below‐described standard of care. All injection procedures were performed with primed needles. Specific neuromodulators used in treating patients included Botox® (Allergan, Irvine CA), Dysport® (Galderma, Fort Worth TX), Xeomin® (Merz, Raleigh, NC), and Jeuveau® (Evolus, Newport Beach, CA). Reconstitution volumes used were for glabellar frown lines (GFL) and lateral canthal lines (LCL) 2.0 mL, and for horizontal forehead lines (FHL) and cheek 5.0 mL for 100 U Vials (Botox, Xeomin, Jeuveau) and 300 U Vials (Dysport). No complications were reported, and the procedures were well tolerated by all patients.

#### 2.3.1 Treatment of forehead horizontal lines (FHL)

Botulinum toxin was injected into the frontalis muscle to treat FHL with the aforementioned 30G, 31G, and 32G needles. The needle was introduced perpendicular to the skin surface and advanced to the intramuscular plane, where toxin was injected in 1–2.5 U onabotulinumtoxinA/incobotulinumtoxinA/prabobotulinumtoxinA aliquots or 3–7.5 U abobotulinumtoxinA aliquots. There was no contact with bone on injection in this area. After three such injections were performed, the needle was removed from the syringe and collected. The same injection technique was used twice more, and two more needles were collected after five and 10 injection passes.

#### 2.3.2 Treatment of glabellar frown lines (GFL)

Botulinum toxin was injected into the glabellar complex (procerus, medial corrugator, lateral corrugator muscles) with the aforementioned 30G, 31G, and 32G needles. The needle was introduced perpendicular to the skin surface while treating GFL and advanced to the intramuscular layer, where toxin was injected in 2.5–5.0 U onabotulinumtoxinA/incobotulinumtoxinA/prabobotulinumtoxinA aliquots or 7.5–15 U abobotulinumtoxinA aliquots. After three such injections were performed, the needle was removed from the syringe and collected. The same injection technique was used twice more, and two more needles of the same gauge were collected after five and 10 injection passes. This entire sequence was repeated for the 30G, 31G, and 32G needles.

#### 2.3.3 Treatment of lateral canthal lines (LCL)

Botulinum toxin was injected into the orbicularis oculi muscle with the aforementioned 30G, 31G, and 32G needles. The needle was introduced at an approximately 30‐degree angle to the skin surface with the bevel up and advanced to the immediately subdermal plane, where toxin was injected in 1–2.5 U onabotulinumtoxinA/ incobotulinumtoxinA/ prabobotulinumtoxinA aliquots or 3–7.5 U abobotulinumtoxinA aliquots. After three such LCL injections were performed, the needle was removed from the syringe and collected. The same injection technique was used twice more, and two more needles were collected after five and 10 injection passes. This entire sequence was repeated for the 30G, 31G, and 32G needles.

#### 2.3.4 Treatment of midface/cheek

Botulinum toxin was injected into the midface/cheek area for skin quality improvement with the aforementioned 30G, 31G, and 32G needles. The needle was introduced approximately parallel to the skin surface with the bevel up and advanced to the intradermal plane, where toxin was injected in 1–2.5 U onabotulinumtoxinA/incobotulinumtoxinA/prabobotulinumtoxinA aliquots or 3–7.5 U abobotulinumtoxinA aliquots creating blebs in the skin that resolved spontaneously within minutes. After three such injections were performed, the needle was removed from the syringe and collected. The same injection technique was used twice more, and two more needles were collected after five and 10 injection passes. This entire sequence was repeated for the 30G, 31G, and 32G needles.

### 2.4 Electron microscopic imaging and analysis

Each collected needle (following the facial injections) was mounted on a scanning EM specimen holder and imaged in a JEOL JSM‐7100F Feld Emission Scanning Electron Microscope at BioNIUM Nanofabrication Facility, University of Miami, Miami, FL, USA. Magnifications are ranging from 100× to 2000×. The microscope was operated at 1 kV, spot size 4, and was maintained at a working distance of approximately 10 mm. Images were then digitally captured as “.tif” files. A total of 45 EM needle tip images were generated.

The EM images produced were processed using the ImageJ photo analysis software (NIH, Bethesda, MD, USA) for subsequent evaluation. Given the differences in image angle, an oval was overlaid on both the internal and external contours of each needle tip outlining the bevel. The diameters of both internal and external circumferences were manually established for each EM image, utilizing the reference standard length present in each image to compensate for variations in image magnification. Both diameters served to compute the effective needle surface area tailored for each EM image. The area of deformation was then calculated as the ratio between intact and deformed needle tip, and the percentage of the respective deformation was calculated to account for different needle sizes (30G, 31G, and 32G).

### 2.5 Statistical analysis

The variables of interest were the percentage of deformation, needle sizes, and number of performed injection passes. The percentage of deformation represents an adjusted value due to its relationship to the needle size: a smaller‐sized needle might display smaller deformation in absolute numbers but might have a larger percentage after adjusting to needle size. Statistical analyses were carried out with SPSS Statistics 25 (IBM, Armonk, NY, USA), and results with a probability level of ≤0.05 were deemed statistically significant.

## 3 RESULTS

### 3.1 General observations

A total of *n* = 45 needle tips were investigated for the purposes of this study. Of those, *n* = 5 were controls (one of each needle size: 30G, 31G, 32G), and *n* = 1 was investigated after touching the inside of the glass vial of the neuromodulator product, and *n* = 1 was investigated after pushing the needle tip through the rubber stopper of the neuromodulator product. Immediately following the injection procedures, visual inspection was performed on all needles by the injectors. No needle tip deformation was appreciated on gross examination.

The relevant size of the needle tip when measured as a 2‐dimensional oval area was for the 30G needle tip 202 819 μm^2^, for the 31G needle tip it was 163 418 μm^2^, and was for the 32G needle tip 138 341 μm^2^.

### 3.2 Investigating “Control” needle tips

The mean percentage of deformation for the control (=unused) needle tips was 4.61% (7.0) [range: 0.40–18.48].

The measured deformation of the control needle passing through the rubber stopper (without any consecutive aesthetic injection) was 5.46%.

The measured deformation of the control needle touching the inside of the glass vial once (without any aesthetic injection into a patient) was 4.36%.

### 3.3 Facial regions injected

**FIGURE 1:**
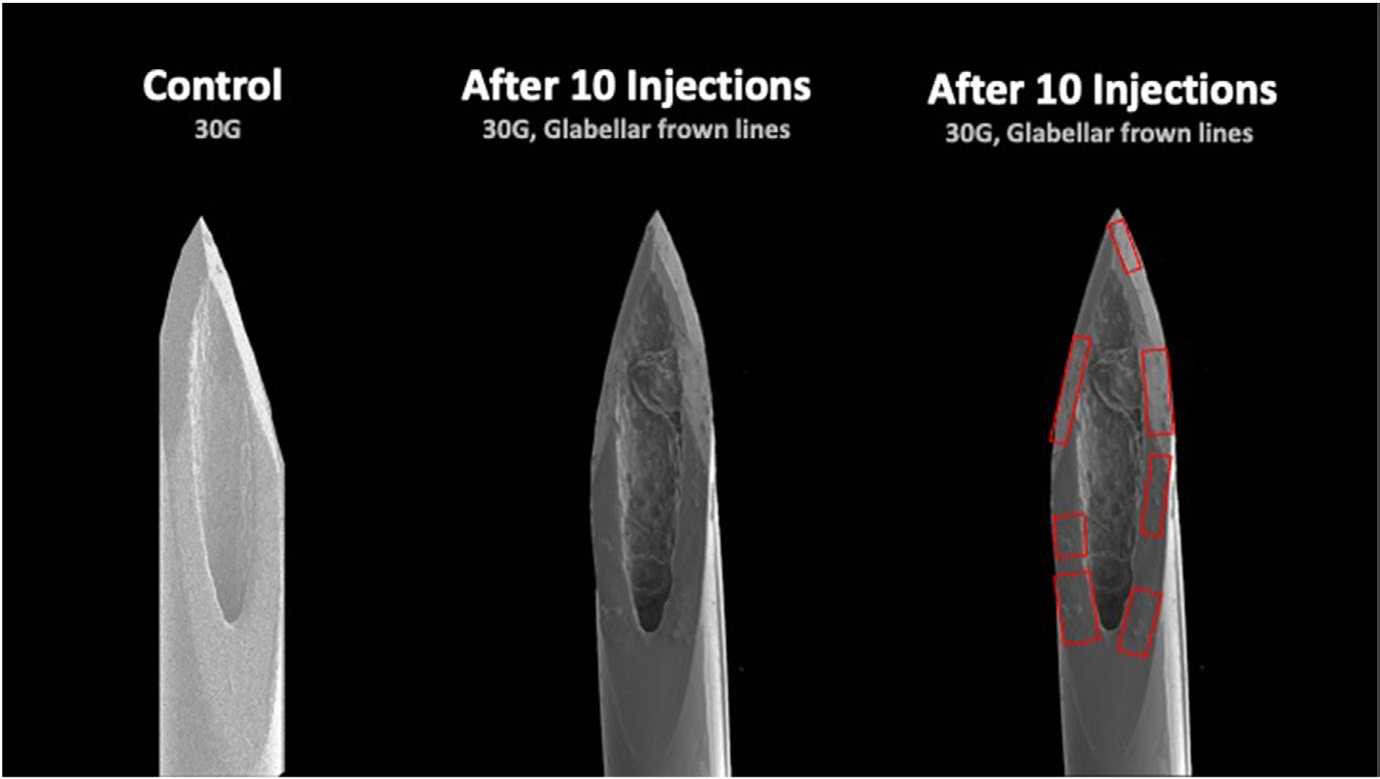
Electron microscopic images of a control 30G needle and a 30G needle after 10 injections into Glabellar Frown Lines. The areas of damage of the needle tip surface are indicated by red boxes.

**FIGURE 2:**
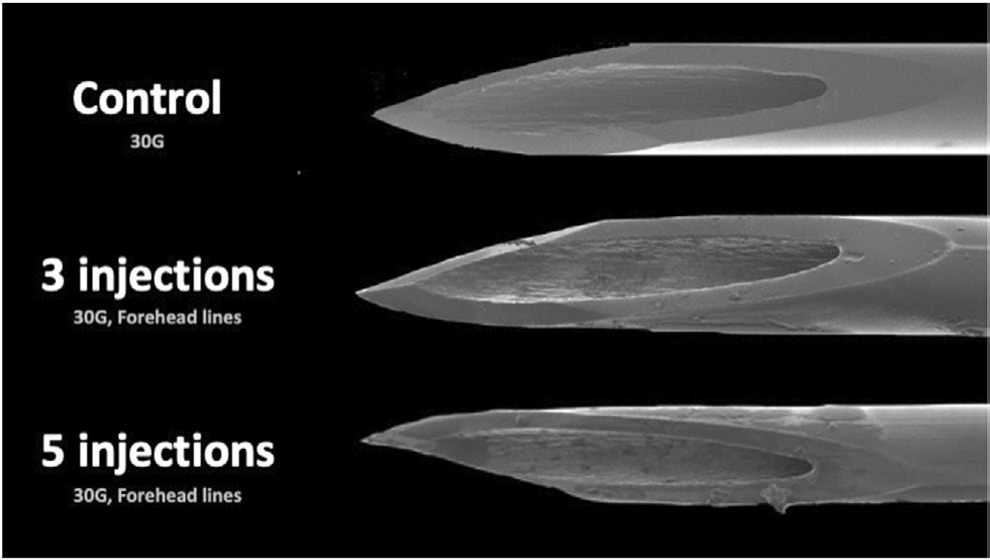
Electron microscopic images of a control 30G needle, a 30G needle after three injections and a 30G needle after 10 injections into Horizontal Forehead Lines.

**FIGURE 3:**
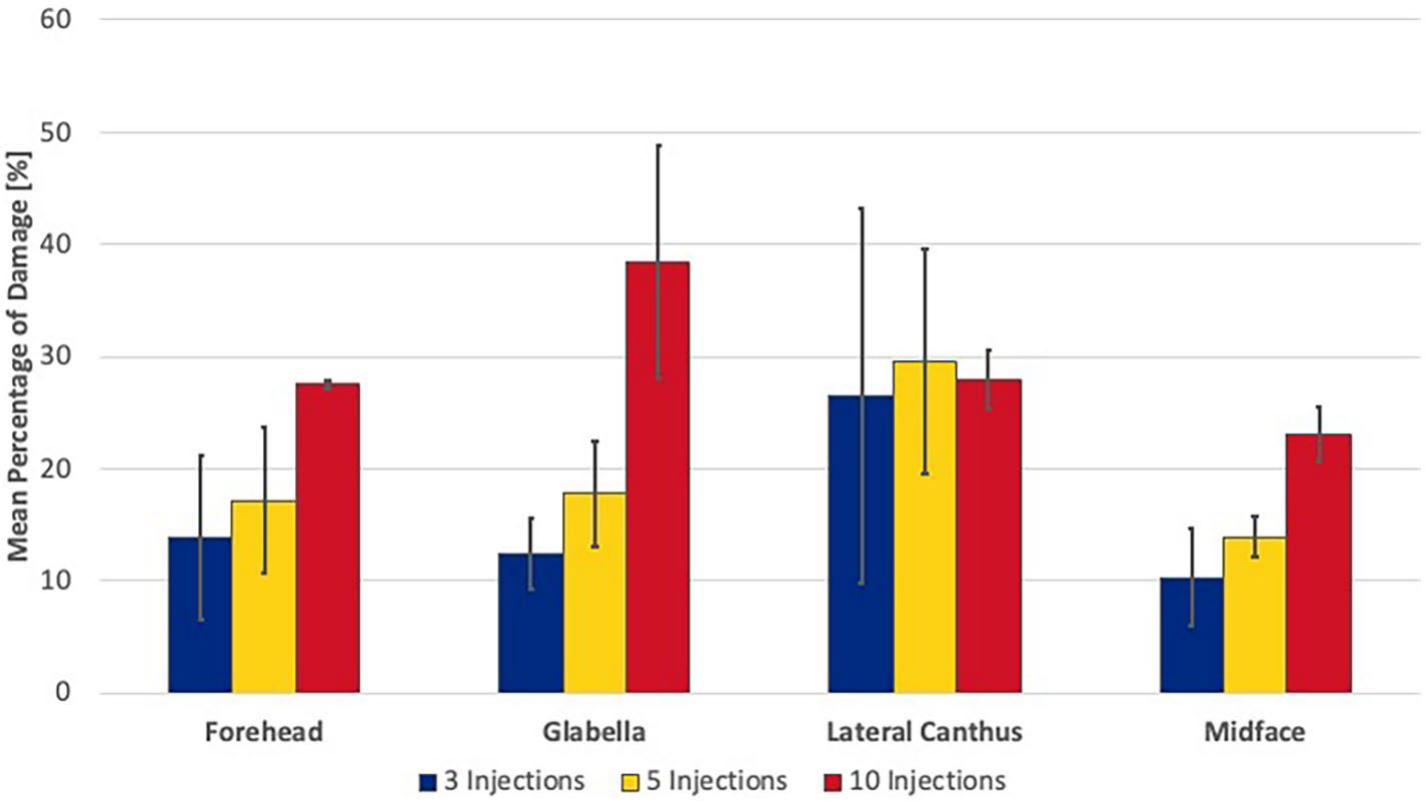
Bar graph the mean percentage of damage of the needle tip surface after three, five and 10 injections for the forehead, glabella, lateral canthus, and midface.

When comparing the various facial regions injected (forehead, glabella, periorbital, and midface) no statistically significant differences in the percentage of deformation were found when injecting 3× (*p* = 0.220) or 5× (*p* = 0.071). At 10× there was a statistically significant difference (*p* = 0.044) with greatest damage displayed after injecting the glabella (38.4% (10.3) [range: 27.7–48.]), followed by periorbital (27.9% (2.6) [range: 20.0–30.9]), forehead (27.5% (0.4) [range: 27.0–27.9]), and midface (23.1% (2.4) [range: 20.9–25.6]) (Figures 1, 2, 3).

### 3.4 Extent of deformation

**FIGURE 4:**
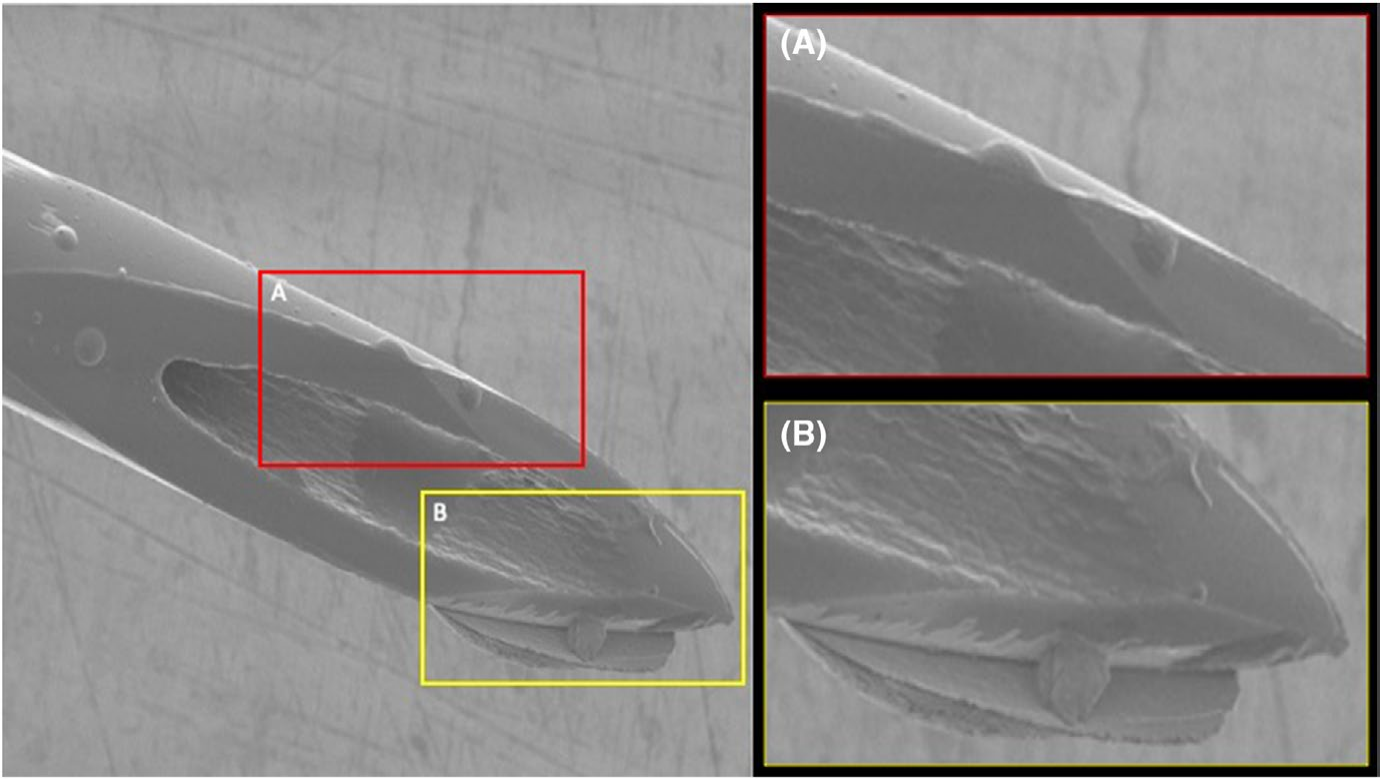
Electron microscopic images of a 30G needle after five injections into midface/cheek.

**FIGURE 5:**
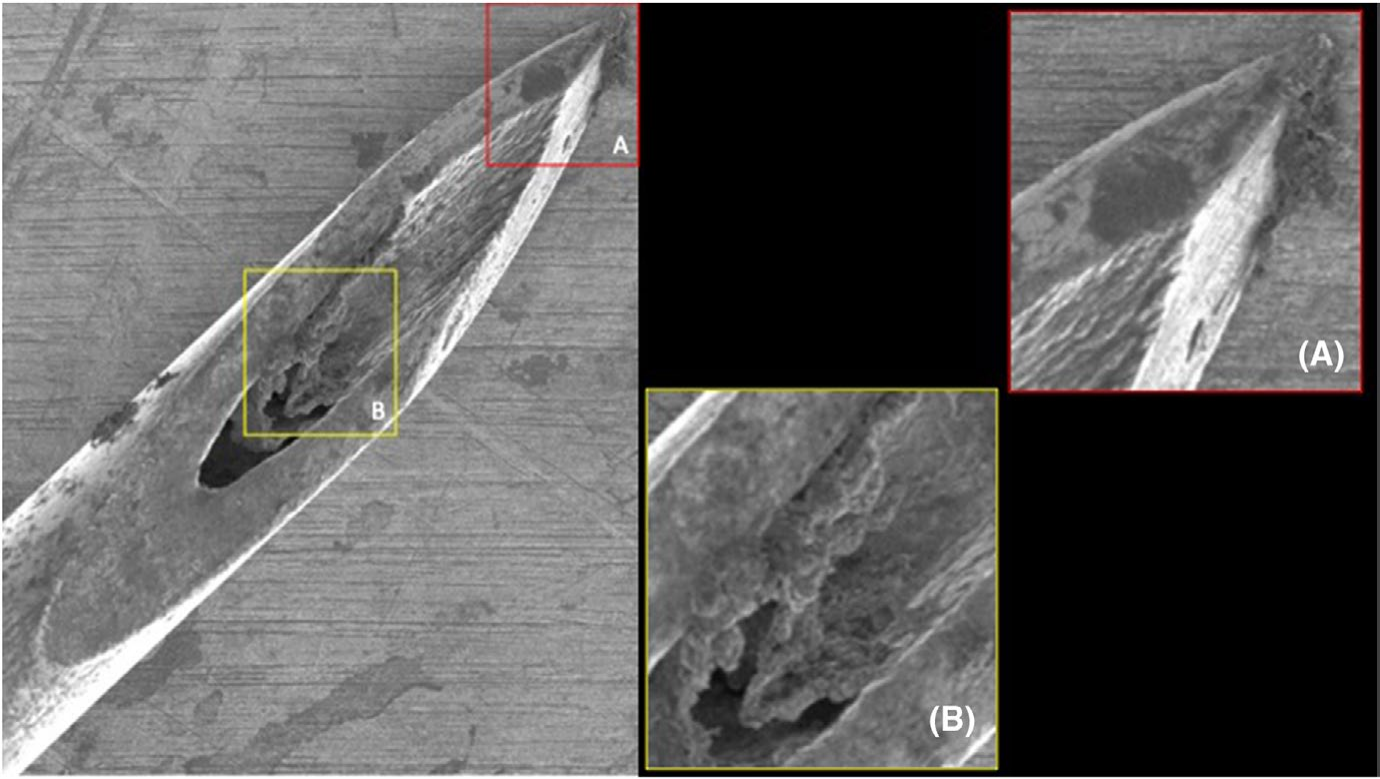
Electron microscopic images of a 32G needle after three injections into Glabellar Frown Lines.

On average and independent of the facial region targeted, the mean percentage of deformation at 3× injection procedures was 14.8% (8.9) [range: 5.8–38.3], at 5× injection procedures 19.6% (8.2) [range: 11.2–40.8] and was at 10× injection procedures 29.2% (7.5) [range: 20.9–48.3] with *p* < 0.001 (Figures 4 and 5).

**FIGURE 6:**
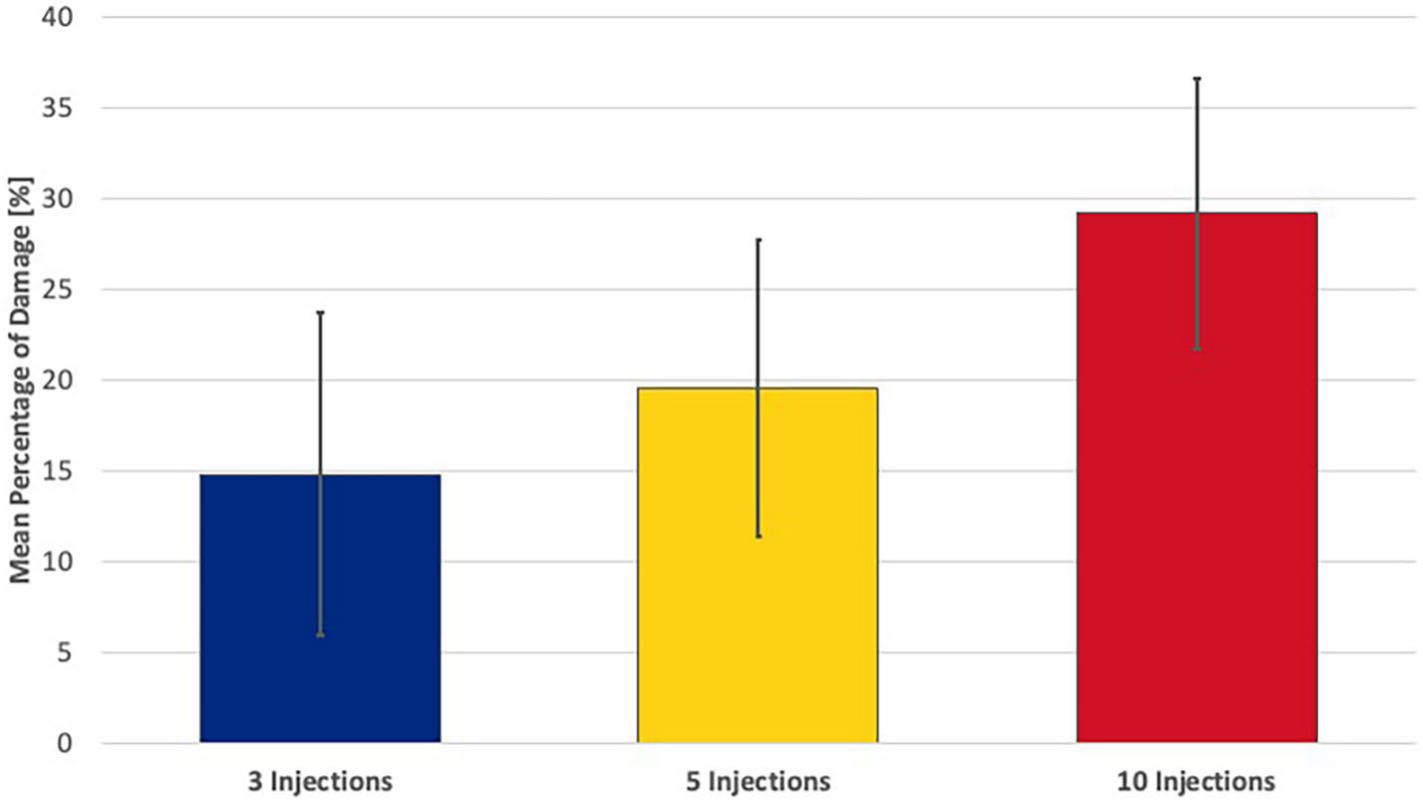
Bar graph of the mean percentage of damage of the needle tip surface after three, five and 10 injections.

**FIGURE 7:**
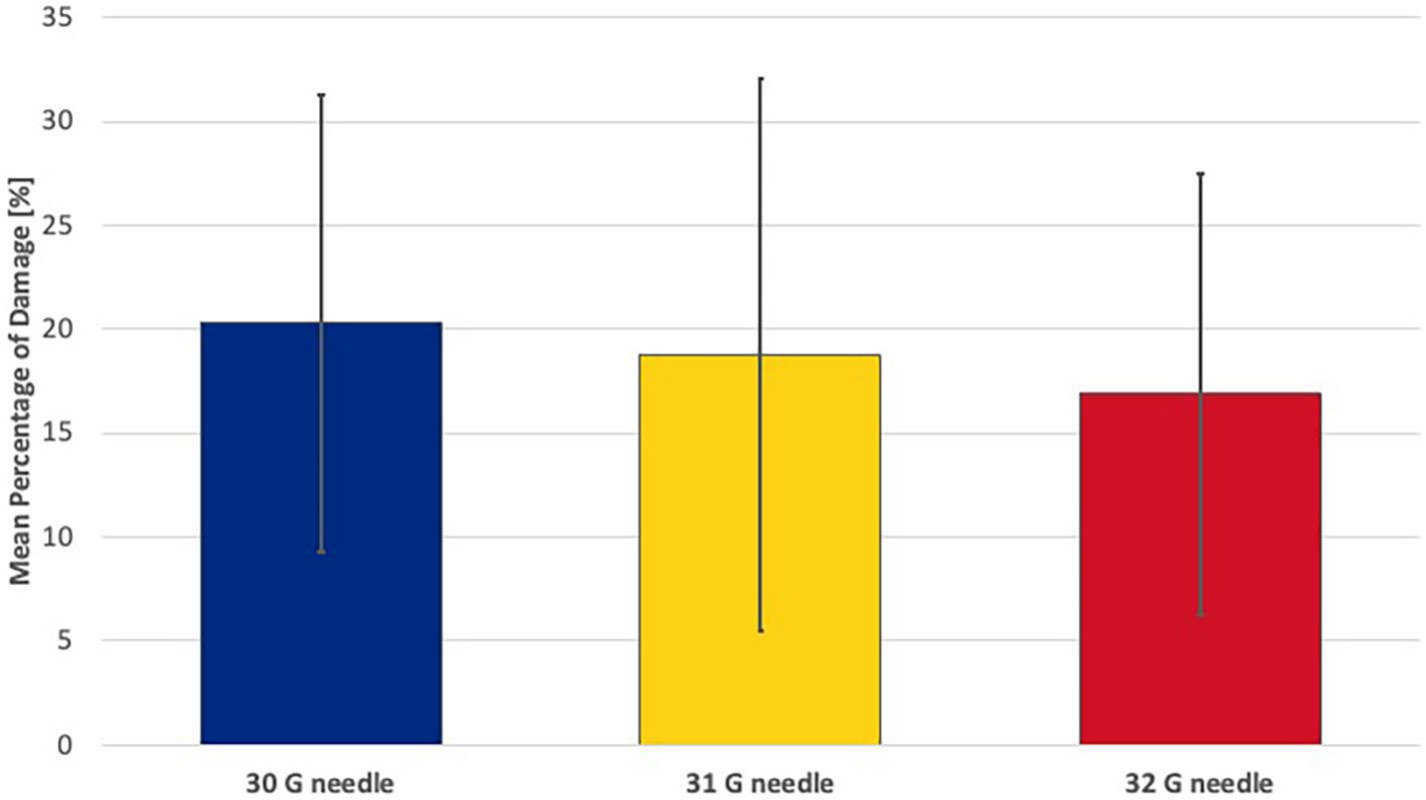
Bar graph of the mean percentage of damage of the needle tip surface for 30G, 31G and 32G needles.

When calculating the numbers of damaged needle tip regions per needle tip area, it was identified that at 3× injections 4.18 (1.4) [range: 2–6] locations, at 5× injections 4.75 (1.4) [range: 2–7] locations, and at 10× injections 5.58 (1.9) [range: 3–10] damaged locations were present with *p* < 0.001 (Figures 6 and 7).

On average, a 30G needle displayed a deformation during the above‐mentioned injections of 20.3% (11.0) [range: 1.0–39.4], whereas a 31G needle displayed deformations of 18.8% (13.3) [range: 3.6–48.3] on average, and a 32G needle displayed deformations of 16.9% (10.6) [range: 0.4–36.5] with *p* = 0.753 for group differences (Figure S1).

When calculating the numbers of damaged needle tip regions per needle tip area, it was identified that a 30G needle showed on average 4.93 (1.8) [range: 1–8] locations, a 31G needle showed on average 4.38 (2.2) [range: 1–10] locations and a 32G needle showed on average 3.64 (1.9) [range: 1–7] locations with *p* = 0.241. (Figure S2).

## 4 DISCUSSION

Electron microscopy imaging has been used to study needles in a variety of medical settings, including acupuncture, dental procedures, and spinal anesthesia.^4^, ^5^, ^6^ To our knowledge, this is the first published study investigating needle deformation following facial neuromodulator injections using EM imaging. Results revealed that irrespective of size, needle tips become increasingly deformed after multiple injection passes. The use of EM allowed for detection of microscopic structural damage to needle tips that could not be appreciated with the naked eye.

Statistical analysis revealed that treating GFL led to the most notable amount of mechanical damage to neuromodulator needle tips. This is likely because treating GFL entails intramuscular injection of botulinum toxin through glabrous skin and into dense muscles. In comparison, treating LCL and FHL involves superficial injections into less substantial muscles, while midface injections for skin quality purposes are placed intradermal.

Regarding needle size, it was observed that the smallest investigated 32G had the least amount of deformation (percentage and damaged regions per surface area), followed by the 31G and the 30G needles. Although this trend did not achieve statistical significance, it is noteworthy, as one would presume needles with the smallest surface area would least likely withstand mechanical damage and shear stress from cutaneous penetration. Injectors should keep this in mind and consider using smaller diameter needles for neuromodulator injections.

As a result of these findings, we recommend injectors performing facial neuromodulator treatments change needle tips after 3–5 injection passes. By changing needle tips, less force will be required to achieve cutaneous penetration potentially leading to increased precision. For safety and efficiency, this likely will require neuromodulator be divided up into several different syringes with attached needles. One could argue for the cost savings associated with using the same needle repeatedly as well as the reduced environmental impact of fewer needles. Despite this, with the magnitude of needle damage as demonstrated by our study, we recommend employing a new needle after 3–5 insertions. The findings of this study are increasingly clinically relevant due to the rise in off‐label neuromodulator treatments for skin quality. Improvements in pore size, oiliness, acne/rosacea, and hyperhidrosis have been reported with multiple small volume (approximately 0.05 mL) intradermal neuromodulator injections placed in a grid‐like fashion over the face.^7^, ^8^, ^9^ None of these treatment algorithms provide guidance for how often an injector should change needles during treatment.

Precision in neuromodulator depth of placement can have an impact on the clinical outcome of the treatment. For instance, one split‐face FHL treatment study showed that injections placed deep intramuscular resulted in more profound effects on forehead movement, whereas neuromodulator placed superficially resulted in less frontalis muscle impact.^10^ The increased force required to introduce a deformed needle into the skin hinders an injector's precision of needle tip placement depth. A deformed needle requiring increased force to achieve cutaneous penetration would be disadvantageous when treating the lateral corrugators for GFL, using a dull needle an injector could inadvertently push the needle deeper and deliver the product to the interdigitating frontalis or levator palpebrae superioris.

It is interesting to note that reference needles already exhibited some deformation without ever being injected into the skin. This is likely due to the process of needle production, shipping, and handling causing some small damage to the needle surfaces. Of course, this is out of the control of medical professionals using these needles; however, it does serve as an additional reason why providers should be mindful of the impact of the number of injections passes on needle integrity.

One limitation of our study is that we utilized several patients to obtain needles instead of performing all injections on a single patient, which would have had the benefit of minimizing variability in skin thickness. This was intentional for the sake of practicality; additionally, injecting and obtaining needles from several patients (as opposed to a standardized laboratory environment) is reflective of daily operations in a high‐volume clinical aesthetic practice. Another limitation of our study is the small number of needles analyzed in our study; this is due to our investigation being entirely self‐funded. A third limitation is the absence of measurement of objective patient pain scores in our study. This is certainly an avenue for future research, as anecdotally with more injection passes with a single needle, patient discomfort increases.

## 5 CONCLUSION

Our study findings support what one would presume—the more times a needle penetrates a patient's skin, the more mechanical damage is caused and the more its material integrity is compromised. To our knowledge, this is the first investigation utilizing EM to provide objective evidence for needle tip deformation after repeated neuromodulator injections. This mechanical damage is greatest when injections are placed intramuscularly and when injections occur more than three times successively. Our goal is for injectors to appreciate the impact of repeat neuromodulator injections on needle deformation and consider the clinical implications of repeated needle use, especially the impact on precision of neuromodulator placement.

## FUNDING INFORMATION

The authors received no financial support for the research, authorship, and publication of this article. The study (imaging, materials used) was entirely self‐funded by the authors.

## CONFLICT OF INTEREST STATEMENT

The authors declared no potential conflicts of interest with respect to the research, authorship, and publication of this article.

## ETHICS STATEMENT

This study did not require ethic board approval due to the study design and the nature of the investigative approach.

## Supporting information


Figures S1–S2.

## Data Availability

The data that support the findings of this study are available from the corresponding author upon reasonable request.
