# Supplementary material for: When to change needles during neuromodulator injections—An electron‐microscopy investigation into needle tip deformation
Source: J Cosmet Dermatol. 2024 Nov 5;24(1):e16506. doi: 10.1111/jocd.16506 (PMC11743232; doi:10.1111/jocd.16506)
Supplement: Supplementary file 1 — Figures S1–S2. [file JOCD-24-e16506-s001.zip › Figure Captions.docx]

Figure S1: Bar graph of the mean number of areas of damage of the needle tip surface after 3, 5 and 10 injections.

Figure S2: Bar graph of the mean number of areas of damage of the needle tip surface for 30G, 31G and 32G needles.
