# Supplementary figures and images for: When to change needles during neuromodulator injections—An electron‐microscopy investigation into needle tip deformation
Source: J Cosmet Dermatol. 2024 Nov 5;24(1):e16506. doi: 10.1111/jocd.16506 (PMC11743232; doi:10.1111/jocd.16506)

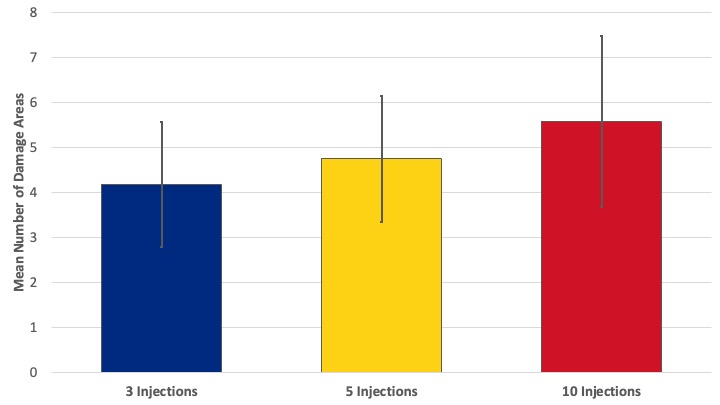

Supplement: Supplementary file 1 — Figures S1–S2. [file JOCD-24-e16506-s001.zip › jocd16506-sup-0001-FigureS1.jpg]

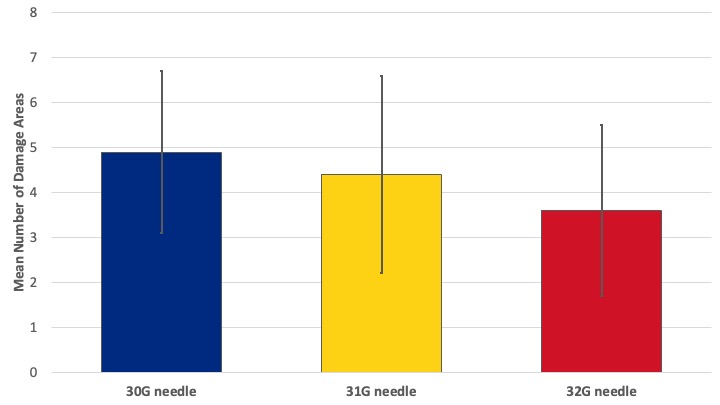

Supplement: Supplementary file 1 — Figures S1–S2. [file JOCD-24-e16506-s001.zip › jocd16506-sup-0002-FigureS2.jpg]
